# Novel humanized anti-PcrV monoclonal antibody COT-143 protects mice from lethal *Pseudomonas aeruginosa* infection via inhibition of toxin translocation by the type III secretion system

**DOI:** 10.1128/aac.00694-24

**Published:** 2024-09-13

**Authors:** Shunsuke Numata, Takafumi Hara, Masaaki Izawa, Yosuke Okuno, Yasuhiko Sato, Shoji Yamane, Hideki Maki, Takafumi Sato, Yoshinori Yamano

**Affiliations:** 1 Laboratory for Drug Discovery and Disease Research, Shionogi & Co., Ltd., Toyonaka, Japan; 2 Shionogi TechnoAdvance Research & Co., Ltd., Toyonaka, Japan; 3 Business Development Department, Shionogi & Co., Ltd., Toyonaka, Japan; Providence Portland Medical Center, Portland, Oregon, USA

**Keywords:** *Pseudomonas aeruginosa*, type III secretion system, PcrV, anti-virulence therapy, monoclonal antibody, mouse infection model

## Abstract

Treatment of *Pseudomonas aeruginosa* infection is challenging due to its intrinsic and acquired antibiotic resistance. As the number of current therapeutic options for *P. aeruginosa* infections is limited, developing novel treatments against the pathogen is an urgent clinical priority. The suppression of virulence of *P. aeruginosa* could be a new therapeutic option, and the type III secretion system (T3SS), which enables the bacteria to translocate various kinds of toxins into host cells and inhibits cellular functions, is considered as one possible target. In this report, we examined T3SS inhibition by COT-143/INFEX702, a humanized monoclonal antibody against PcrV, T3SS component, and present the crystal structure of the antibody-PcrV complex. COT-143 inhibited T3SS-dependent cytotoxicity and protected mice from the mortality caused by *P. aeruginosa* infection. The inhibition of cytotoxicity coincided with inhibition of translocon formation in a host cell membrane, which is necessary for T3SS intoxication. COT-143 protected murine neutrophils and facilitated phagocytosis of *P. aeruginosa*. These results suggest that COT-143 facilitates *P. aeruginosa* clearance by protecting neutrophil via inhibition of T3SS-dependent toxin translocation. This is the first report to show that an anti-PcrV antibody directly interferes with translocon formation to inhibit intoxication of host cells.

## INTRODUCTION

*Pseudomonas aeruginosa* is one of the most prevalent etiological agents in healthcare-associated infections, especially in lower respiratory tract infections. As *P. aeruginosa* has frequently intrinsic and acquired antibiotic resistance, there are limited options for antibiotic treatment. A concern is highlighted by the World Health Organization by classifying multi-drug resistant *P. aeruginosa* as a serious threat to human health. A versatile set of virulence factors (e.g., surface appendages such as type IV pili and secreted factors including proteases and exopolysaccharides) is thought to contribute to host immune avoidance and adaptation to the host (infection site)-specific environments including exposure to antibiotics (1, 2). While therapeutic agents inhibiting those factors may improve *P. aeruginosa* infection outcomes, targeting one specific factor may have a very limited effect. To this end, protein secretion systems which govern various kind of virulence factors at the same time are thought to be a good candidate.

The type III secretion system (T3SS) is a protein translocation mechanism used by different kinds of pathogenic bacteria to inhibit and/or control host cellular functions to avoid attack from the host’s defense systems and facilitate bacterial dissemination in the host (3–7). The T3SS of *P. aeruginosa* consists of a syringe-like structure with needle (8). Various proteinaceous toxins called effectors are delivered from the bacterial cytosol to that of the target cell through the structure and inhibit cellular biochemical and enzymatic reactions or disrupt structures needed to invoke cellular defensive reactions to bacterial invasion (8). In *P. aeruginosa*, hydrophobic PopB and PopD proteins interact with the host cell membrane to form a small pore. The approximate internal and external diameters of the pore reported as 3–4 and 8–10 nm reported by Gébus et al. (9), then the needle tip is connected to the pore to inject effectors into the host cell cytosol (10–16). The T3SS is activated by various stimuli, such as contact between bacteria and the target host cell, and low calcium concentration (17–19). Four effectors are identified so far in *P. aeruginosa*, ExoS, ExoT, ExoY, and ExoU. ExoS, and ExoT, which share high sequence identity, are bifunctional enzymes with amino-terminal Rho-GAP and C-terminal ADP-ribosyltransferase activities. ExoS inhibits phagocytosis and reactive oxygen species production by neutrophils, and induces apoptosis, while ExoT inhibits host cell division and induces mitochondrial apoptosis (20–23). ExoU, phospholipase A, is anchored on the inner surface of cell membranes after translocation into target cells and causes cell lysis (24–26). The function of ExoY has not been studied well, but a relatively minor role in an infection is suggested (27). Although the precise mechanism for the T3SS activation is largely unknown, PcrV, the needle-tip protein located on top of the needle, is known to be essential for the activation and regulated delivery of those effectors (7, 13, 19, 28, 29). With its central role in T3SS regulation, PcrV is known to be abundant in the blood of patients with *P. aeruginosa* infections and has been suggested to be a therapeutic target (28, 30, 31), and many attempts to create anti-PcrV antibody for therapeutic purpose were reported (32–36). COT-143 is a novel humanized anti-PcrV monoclonal antibody originally created by Shionogi & Co., Ltd., and is undergoing a first-in-human clinical trial as a treatment for the reduction of severity and frequency of exacerbations in patients with non-cystic fibrosis bronchiectasis colonized with *P. aeruginosa* by Infex Therapeutics as INFEX702 (https://www.infextx.com/infex-therapeutics-to-begin-clinical-development-of-anti-virulence-therapy-for-pseudomonas-aeruginosa-pa-in-non-cystic-fibrosis-bronchiectasis-patients/).

In this study, we investigated the therapeutic effect of COT-143 in a murine *P. aeruginosa* lung infection model. We also investigated the mechanisms by which COT-143 inhibits *P. aeruginosa* virulence including a crystallographic analysis.

## RESULTS

### COT-143 inhibited cytotoxicity of *P. aeruginosa*

**Fig 1:**
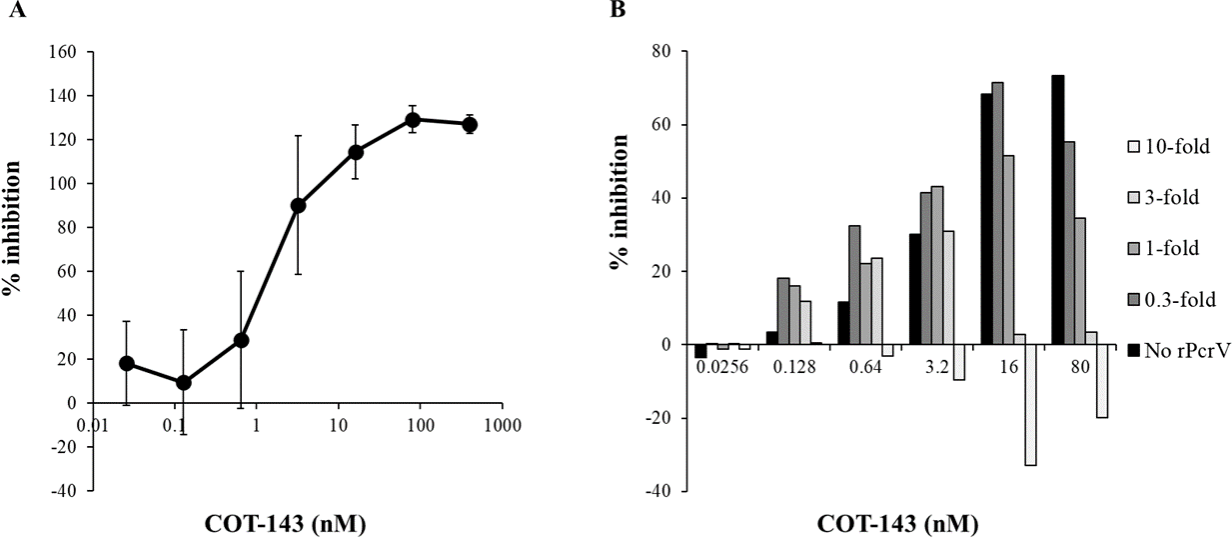
*in vitro* cytotoxicity inhibitory activity of COT-143 against human cells. (**A**) Cytotoxicity inhibitory activity (%) of COT-143 in co-culture system of U937 (human lymphoma cell line) and *P. aeruginosa* PA103 is shown. The cytotoxicity was determined by the measurement of lactose dehydrogenase released from U937 cells. (**B**) Neutralization effect on the cytotoxicity inhibitory activity of COT-143 in the co-culture system is shown. COT-143 and recombinant PcrV protein (rPcrV) were mixed and pre-incubated at room temperature for more than 15 min. The rPcrV amount was 0.3, 1.0, 3.0, and 10.0-fold that of tCO-143 in molar ratio.

The cytotoxicity of *P. aeruginosa* PA103 in the U937 cell system was inhibited by COT-143, with an IC_50_ of 2.8 nM, in a dose-dependent manner (Fig. 1A). The cytotoxicity inhibitory activity of COT-143 at the concentration of 80 nM was suppressed by purified recombinant PcrV protein (rPcrV) in a dose-dependent manner (Fig. 1B). The cytotoxicity inhibitory activity was completely neutralized by 10-fold rPcrV at all the concentrations of COT-143 tested. The T3SS dependency of the cytotoxicity of PA103 was confirmed in *pcrV*- and *exoU*-deletion mutants (data not shown).

### Crystal structure of the h1F3 Fab-PcrV complex

**Fig 2:**
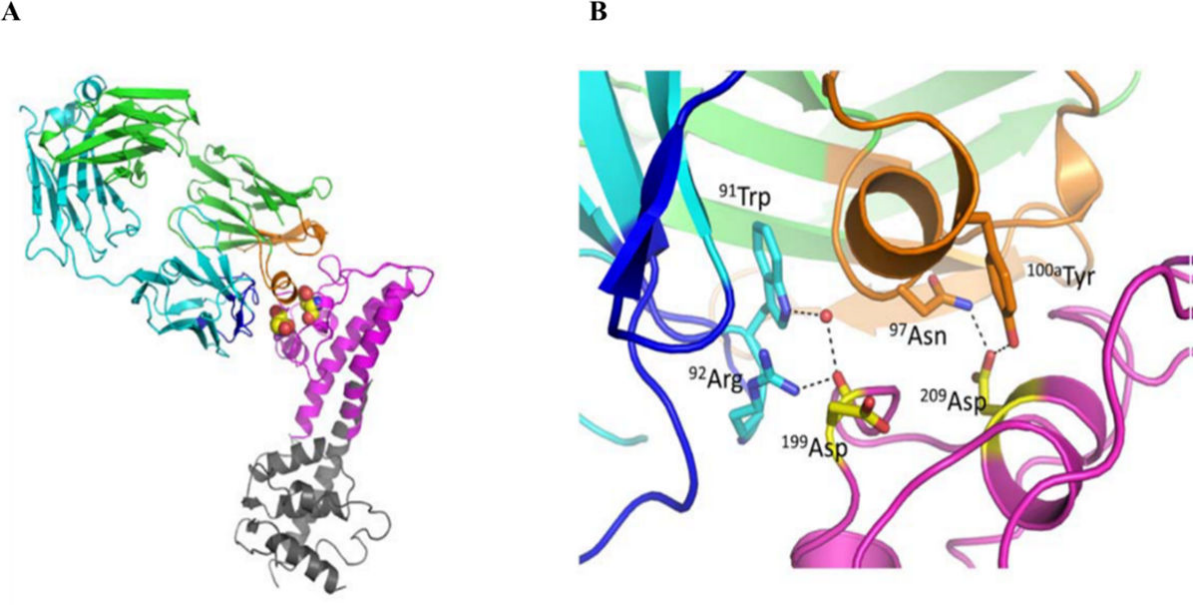
A crystal structure of the complex between h1F3 Fab and PcrV (130–263): interactions among complementarity-determining region (CDR) heavy chain 3, CDR light chain 3, and two key Asp residues of PcrV. Heavy and light chains of the h1F3 are in green and cyan. The CDR heavy chain and CDR light chain of COT-143 are emphasized by orange and blue. The partial PcrV (130–263) is in magenta. The other region of the PcrV structure (gray) is drawn by using a crystal structure of LcrV (PDB ID: 1R6F), which is a PcrV homolog of *Yesrsinia pestis*. The two PcrV residues of ^199^Asp and ^209^Asp are highlighted in yellow. (**A**) Whole structure of h1F3 Fab and partial PcrV (130–263) complex. (**B**) Amino residue interactions between h1F3 Fab and PcrV (130–263).

A crystal structure of the complex between h1F3 Fab (pre-optimized COT-143 that has a single amino acid change in the framework region of the light chain of COT-143) and PcrV (130–263) (at the 130th–263rd positions) was solved at 2.0-Å resolution (Fig. 2). The high-resolution X-ray data clarified the structural basis of the antibody-antigen interaction, including water molecules. The epitope residues in PcrV were identified in the positions of ^163^Arg to ^170^Asp, ^197^Asn to ^209^Asp, and ^226^Asp. The interactions between residues in CDR3 of h1F3 and PcrV are listed in Table S1.

### COT-143 protected mice from *P. aeruginosa* lung infection

**Fig 3:**
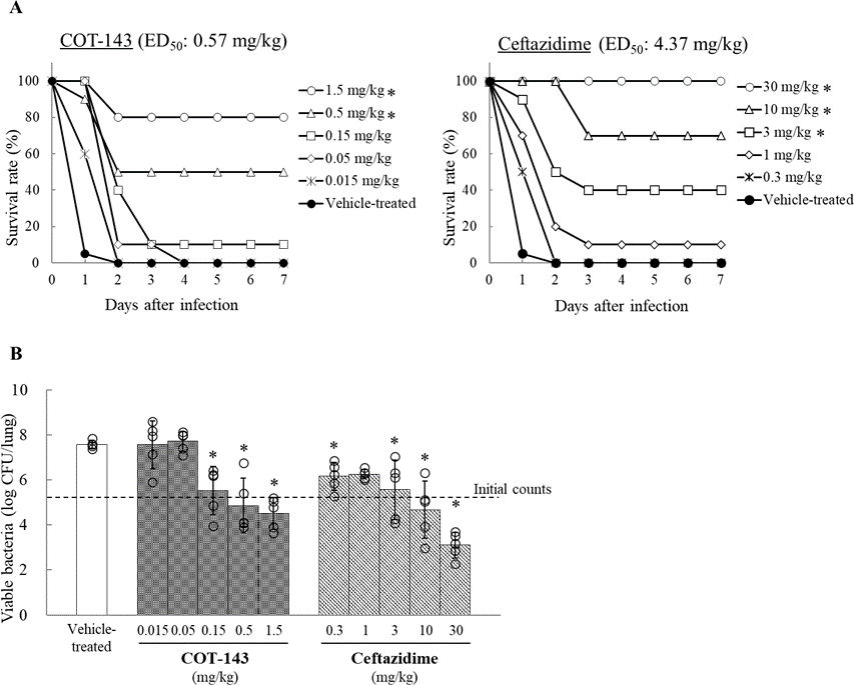
Effect of COT-143 on mouse survival rate and pulmonary growth of *P. aeruginosa* in a pneumonia model. (**A**) Mouse survival rate (%) 7 days after infection (10 mice/group except for the vehicle-treated group, where 20 mice were used) is shown. Effective dose for 50% survival 7 days after infection (ED_50_) in each regimen (time of administration) was estimated by logistic regression model. *P < 0.05 vs vehicle-treated group (Fisher’s exact test). (**B**) Viable bacterial counts in lungs in each group 24 hours after infection (log_10_ CFU/lung) are shown. Each circle represents individual bacterial count, and the bar represents the mean of counts in each group (five mice/group). *P < 0.05 vs vehicle-treated group (Dunnett’s multiple comparison test).

Treatment with COT-143 by a single intravenous administration or ceftazidime by a single subcutaneous administration increased survival rates of murine *P. aeruginosa* lung infection dose-dependently (Fig. 3A). A significant increase in the survival rate was observed in groups treated with ≥0.5 mg/kg of COT-143 and ≥3 mg/kg of ceftazidime, respectively. The efficacy of ceftazidime suggests that the model is representative of a clinical setting where antibiotic treatment takes place. The 50% effective dose of COT-143 and ceftazidime was 0.57 and 4.37 mg/kg, respectively.

Twenty-four hours after infection, a dose-dependent decrease of bacterial cells in the lungs was observed in the COT-143-treated group, as well as in the ceftazidime-treated group (Fig. 3B). COT-143 at doses of ≥0.15 mg/kg resulted in a significant decrease of viable bacteria cells in lungs compared to the vehicle-treated group.

Ceftazidime at ≥0.3 mg/kg showed a significant decrease of bacteria cells in the lung compared with the vehicle-treated group.

### COT-143 affected cytokine levels in the lungs of mice with *P. aeruginosa* lung infection

**Fig 4:**
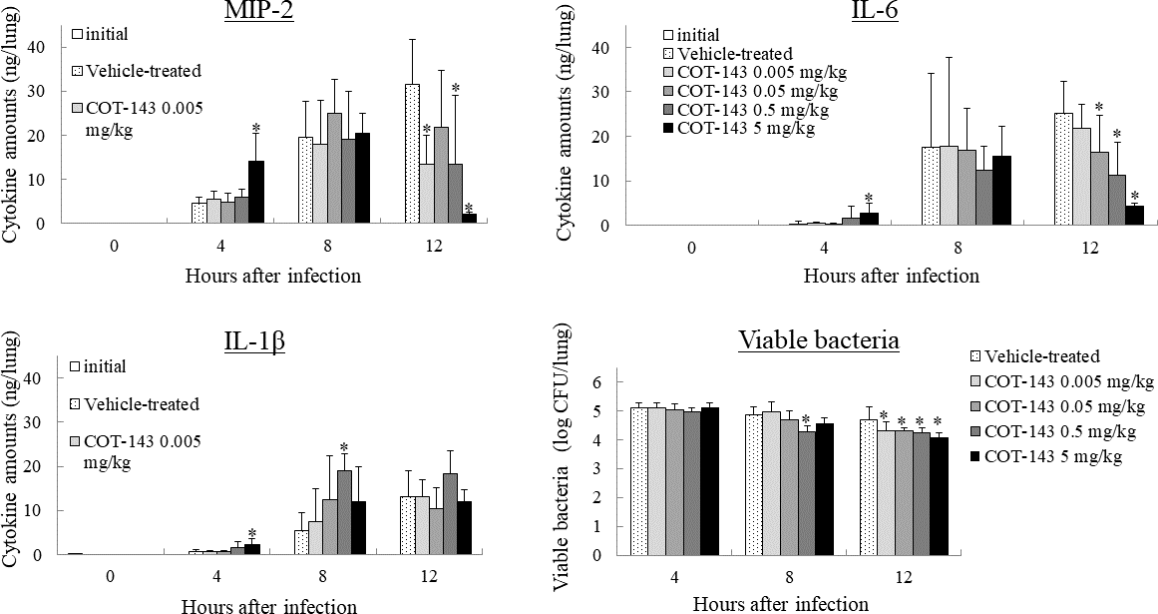
Effect of COT-143 on pulmonary cytokine levels and number of viable bacteria in a mouse pneumonia model. Cytokine levels (ng/lung) and viable bacterial counts (log_10_ CFU/lung) in lungs in each group 4, 8, and 12 hours after infection are shown (eight mice/group). Cytokine levels in non-infected animals are also shown ( three mice/group). *P < 0.05 vs vehicle-treated group (Dunnett’s multiple comparison test). “Initial” corresponds to the cytokine levels in non-infected animals.

At 4 and 8 hours after infection, levels of pulmonary macrophage inflammatory protein (MIP)-2, interleukin (IL)-6 and IL-1β were increased in the COT-143-treated and vehicle-treated group. At 12 hours after infection, pulmonary levels of MIP-2 and IL-6 decreased in mice treated with 0.5 and 5.0 mg/kg of COT-143. On the other hand, the level of IL-1β did not decrease in COT-143-treated groups at any measurement time. The viable bacterial counts in lungs were almost the same among each of the COT-143-treated groups and the vehicle-treated group (Fig. 4).

### COT-143 inhibited contact-dependent hemolysis and T3SS translocon insertion

**Fig 5:**
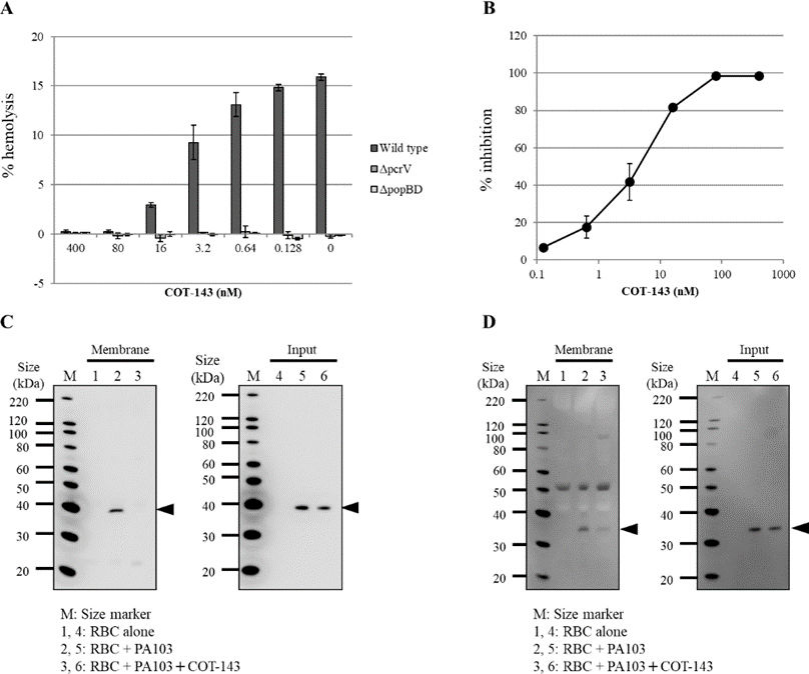
Effect of COT-143 on contact-dependent hemolysis and T3SS translocon insertion. (**A**) Hemolysis levels in co-culture system of sheep red blood cells (RBCs) and *P. aeruginosa* PA103 with various concentrations of COT-143 are shown. Hemolysis was determined by measuring hemoglobin released from RBC. Isogenic *pcrV* and *popBD* deletion mutant of PA103 were used as a negative control. (**B**) Hemolysis inhibitory activity of COT-143 is shown. (**C and D**) Effect of COT-143 on the localization of translocon components in RBC membranes is shown. RBC membrane after the contact with PA103 with or without COT-143 (400 nM) was fractionated by sucrose-gradient ultracentrifugation, and the amount of PopB protein (**C**) and PopD protein (**D**) was determined by Western blotting using anti-PopB and anti-PopD polyclonal antibodies, respectively. Input control represents the PopB and PopD amount before the fractionation. Theoretical molecular weights of PopB and PopD are 40.1 and 31.3 kDa, respectively.

*P. aeruginosa* uses T3SS to cause hemolysis by contact-dependent pore formation in red blood cell (RBC) membranes (13, 14). The pore, called translocon, consists of two hydrophobic proteins, PopB and PopD. We examined the effect of COT-143 on the contact-dependent hemolysis and translocon formation. COT-143 inhibited lysis of RBC in a dose dependent manner (Fig. 5A and B). IC_50_ of the inhibition was 4.2 nM. As a control, both *pcrV*-deleted and *popBD*-deleted mutants showed no hemolytic activity. Examination of the amount of PopB and PopD in the RBC membrane fraction by Western blotting analysis revealed that COT-143 decreased the amount of both proteins (Fig. 5C and D). These results suggest that COT-143 protects RBC from lysis by inhibiting the contact-dependent translocon formation in the membrane.

### COT-143 enhanced bacterial phagocytosis by murine neutrophil

**Fig 6:**
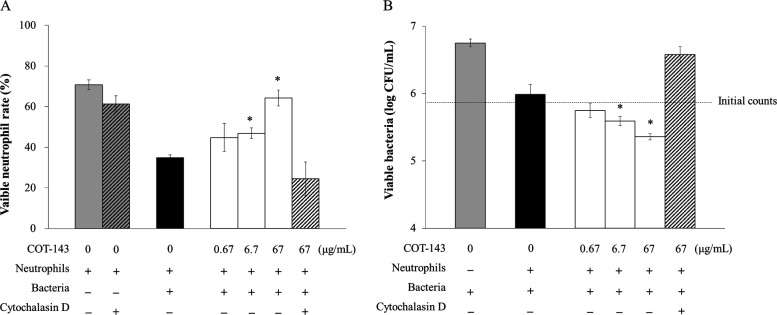
Effect of COT-143 on bacterial phagocytosis by murine neutrophil. (**A**) Viable neutrophil rate (%) in a co-culture system of murine primary neutrophils and *P. aeruginosa* PA103 with various concentrations of COT-143 is shown. Cytochalasin D (0.5 µg/mL) was added to inhibit phagocytosis by neutrophils. (**B**) Viable bacterial count (CFU/mL) in the co-culture system in panel** A** is shown.

Neutrophils are the first-line immune arsenal to eliminate bacteria in an acute lung infection. After incubation of *P. aeruginosa* PA103 with mouse neutrophils, neutrophil survival rate and number of viable bacteria decreased simultaneously. COT-143, at the concentration of ≥6.7 nM, demonstrated prevention of neutrophil killing and concentration-dependent enhancement of bacterial clearance (Fig. 6). These activities of COT-143 were fully antagonized in the presence of cytochalasin D, an inhibitor of phagocytosis.

## DISCUSSION

The *in vivo* efficacy study has shown that COT-143 protected mice from the T3SS-mediated effects of *P. aeruginosa* lung infection. The protection was associated with inhibition of bacterial growth and suppression of pro-inflammatory cytokine production in the lungs. These results indicate that COT-143 has potent inhibitory activity against *P. aeruginosa*-induced inflammation and mortality in a murine model of infection. The *in vitro* cytotoxicity of *P. aeruginosa* against human cells was inhibited by COT-143 in a dose-dependent manner, and the inhibitory activity was completely antagonized by addition of recombinant PcrV protein, suggesting that COT-143 was absorbed by recombinant PcrV. Previous reports show that a well-characterized anti-PcrV antibody Mab166, a base antibody for KB001 an anti-PcrV PEGylated monoclonal antibody fragment clinical development, exhibited cytotoxicity inhibitory activity with an IC_50_ of 2.8 µg/mL and provided mouse protection in an acute lung infection model at the dose range of ≥5 mg/kg (36–39). In comparison with Mab166, the present study revealed that COT-143 exhibited ≥5-fold lower IC_50_ in a cell-based assay and provided significant protection at a lower dose range (≤0.5 mg/kg) in a mouse infection model. When mapped onto the modeled PcrV structure, the epitopes of Mab166 and KB001 are located in close proximity to the pore formed by the PcrV multimer, while the epitope of COT-143 is located in a similar region but more distal to the pore (data not shown). We also confirmed that the Fab form of COT-143 lost anti-virulence activity, while those of Mab166 and KB001 retained the activity (data not shown). These data suggest that COT-143 sterically inhibit PcrV function to insert PopB and PopD, while Mab166 and KB001 may act in a bulkiness-independent manner such as pore occlusion.

COT-143 inhibited contact-dependent hemolysis and decreased insertion of translocon components into RBC membranes. These results suggest that COT-143 suppresses cytotoxicity of *P. aeruginosa* via inhibition of PcrV-dependent translocon insertion into a host cell membrane and leads to decreased toxin translocation. This is the first report to confirm the necessity of PcrV protein for intramembrane assembly of both PopB and PopD proteins, pharmacologically. *P. aeruginosa* spp. lacking *pcrV* are still able to secrete PopB and PopD but not to insert them into a host cell membrane, suggesting that PcrV acts as a molecular chaperone facilitating pre-insertion conformation of the highly hydrophobic PopB-PopD protein (13). Our results suggest that COT-143 binding alters the interaction between PcrV and the two translocon components to abolish the chaperon-like function of PcrV, which resulted in loss of translocon formation. As the precise mechanism for PopB-PopD assembly in host cell membranes is still not clear, our findings shed new light on the molecular mechanism for PcrV-assisted translocon formation. The presented structural analysis of the complex between the h1F3 Fab and PcrV (130–263) complex reveals that COT-143 binds to a top part of PcrV, which is thought to be an interface for the interaction between PcrV and translocon components. This supports the above-mentioned model where COT-143 interferes with the interaction between PcrV and translocon components to inhibit chaperon function of PcrV required for the insertion of the translocon. Finally, we have shown that COT-143 enhanced bacterial phagocytosis in a co-culture system of *P. aeruginosa* and mouse neutrophils. A previous study has also shown that an anti-PcrV antibody increased the number of *P. aeruginosa* phagocytosed by neutrophils in lungs of infected animals (40). These results suggest that inhibition of *P. aeruginosa* virulence by COT-143 contributes to retention of neutrophil phagocytic function, thus enhancing bacterial clearance, which results in host cell protection from lethal *P. aeruginosa* infections.

*P. aeruginosa* is a commensal bacterium causing various kinds of infections as an opportunistic pathogen. In many chronic respiratory infections, like cystic fibrosis, non-cystic fibrosis bronchiectasis, and chronic obstructive pulmonary disease, the bacterium has been proven to play important roles in developing and exacerbating patient symptoms (41–45). The T3SS injects various kinds of effectors into host cells to inhibit cellular functions (8), resulting in dysregulated bacterial expansion inside the host’s body. In accordance with this function, high prevalence of T3SS-positive phenotype of *P. aeruginosa* isolated from patients with acute infections, such as hospital-acquired pneumonia, has been reported, and the relationship between T3SS activity and disease severity has been suggested (46–50). The protection of host cells and reduced inflammatory responses in animals by COT-143 suggests that the antibody has a potential to reduce the burden of *P. aeruginosa* infections and reduce the duration or frequency of severe symptoms of chronic *P. aeruginosa* infections such as acute pulmonary exacerbation. Recently, Neupane et al. reported that alveolar macrophages “conceal” infecting bacteria from an inappropriate inflammatory response resulting in lower neutrophil recruitment and limited production of chemokines and pro-inflammatory cytokines such as MIP-2, keratinocyte chemoattractant, and IL-6 (51). Considering their findings, the reduced amount of MIP-2 and IL-6 observed in COT-143-treated animals in this report suggests that inhibition of T3SS contributes to the protection of local immune cells including alveolar macrophage and confers a successful local immune response. While the unchanged IL-1β level suggests basal immune response, low IL-6 induction indicates limited activation of the cytokine. Although many previous reports have shown inhibitory activity of anti-PcrV antibodies on T3SS-dependent intoxication, the molecular basis for the activity was not reported (35, 36, 40, 52, 53). The presented study is the first report on a molecular-level mode of action for an anti-PcrV antibody. The mechanism is a highly effective way to inhibit T3SS by interfering with the crucial step of host cell intoxication and translocon formation, leading to successful host protection from the bacterial virulence. As the mode of action of COT-143 would not interrupt the efficacy of other antibacterial drugs, it would be a novel therapeutic and prophylactic option to reduce infective exacerbations and enhance the efficacy of current therapies for difficult-to-treat chronic *P. aeruginosa* infections that, if uncontrolled, result in reduced quality of life for patients and a higher hospitalization and mortality risk.

## MATERIALS AND METHODS

### Bacterial strains, cells, and medium

Standard strain *P. aeruginosa* PA103 (ATCC29260) was purchased from American Type Culture Collection. PA103 has functional T3SS loci producing three effectors: ExoU, ExoT, and ExoY. The minimum inhibitory concentration of ceftazidime against PA103 was 1 µg/mL in broth microfluidic dilution method (CLSI guideline M07-Ed11) (54). The bacterial stocks were prepared by the following method: PA013 was cultured overnight with shaking at 37°C in Luria-Bertani (LB) [Becton, Dickinson and Company (BD)] broth or Mueller-Hinton Broth (MHB) (BD), mixed to give a 10% glycerol solution and finally frozen at −80°C. The test strain was cultured overnight with shaking at 37°C in LB broth or MHB. The bacterial suspension was diluted with medium or Dulbecco’s phosphate-buffered saline (D-PBS) (Sigma-Aldrich). Gastric mucin was added to the suspension when used in animal infection models with a final concentration of 0.5%.

### Gene deletion of *popBD* in *P. aeruginosa*

The procedure described below is mainly based on the previously reported protocol (55). To construct isogenic mutants lacking popBD, 1-kb region upstream of popBD was amplified from PAO1 chromosomal DNA using delta_BD_1 F#ER (GACGGGAATTCGGCAAGCGCAAGGCGCT) and delta_BD_1 R#Hd (GACTAAGCTTTCAAGCGTTATCGGATTCATATG). Each primer pair has a newly added restriction site, EcoRI and HindIII. A 1-kb region downstream of the popBD was amplified by PCR using the primer pair delta_BD_2 F#Hd (CCTGAAGCTTGTCCAGGTGCGCCAGGGC) and delta_BD_2 R#Bm (CACTGGATCCTCACCTGCGCCGGACCGT). Each primer pair has a newly added restriction site, HindIII and BamHI. The PCR-amplified DNA fragments were digested with corresponding restriction enzymes and ligated into the EcoRI-BamHI site in a multicloning site of pNOT322 (56) to yield pAPV15. A kanamycin resistance cassette was amplified from pBSL128 by PCR using Km cassette_Hd#F (GCCCAAGCTTAATTAATTAACTACTGGGCTATCTGGACAAG) and Km cassette_Hd#R (GCCCAAGCTTAATTAATTAATCAGAAGAACTCGTCAAGAAGG). The kanamycin resistance cassette was digested with MluI and ligated into the HidIII site of pAPV15. After NotI-flanked Mob cassette from pMT5071 (57) was ligated, the resulting plasmid was mobilized from an *E. coli* strain S17-1 (58) to the P. aeruginosa PA103 to introduce deletion of *popBD* into the recipient chromosomes by homologous recombination. The cell mixture was mated on MHA at 37℃ for 3 h and then suspended in saline. The suspensions were plated on minimal agar plates supplemented with kanamycin and incubated at 30℃ for 2 days to isolate kanamycin-resistant strain. The obtained transconjugants were subsequently grown on drug-free MHA overnight for a second homologous recombination and then plated on MHA supplemented with kanamycin and 10% (wt/vol) sucrose. The *popBD* deletions in selected kanamycin-resistant and sucrose-resistant clones were confirmed by Sanger sequencing (data not shown).

### Preparation of recombinant PcrV protein

The recombinant PcrV protein was expressed by *Escherichia coli* BL21(DE3)/pLysS as C-term 6× poly His form and was purified with a Ni-NTA Superflow (GE Healthcare) column. The eluted fraction was further purified by gel filtration chromatography with a Superdex 75 column in Tris-buffered saline (TBS, 50-mM Tris, and 150-mM NaCl; pH 7.5).

### Preparation of COT-143

Mouse anti-PcrV antibody candidates were generated by hybridoma technology after immunization with purified recombinant PcrV. By robust screening, mouse anti-PcrV antibody m1F3 was selected as the best antibody to have the most potent binding affinity to PcrV and protective activity against the killing of mammalian cells among antibody candidates. This antibody was humanized by the complementarity-determining region grafting method with H93 and H94 back-mutation and synthesized as human IgG4Pro format, named h1F3. h1F3 contains an amino acid residue of the mouse sequence, which was modified to the corresponding residue of the human sequence (L128: Ala > Thr), named COT-143.

### X-ray crystallographic analysis

#### Preparation of recombinant PcrV (130–263)

Recombinant protein of PcrV, corresponding to amino acid residues 130Ala-263Asn, was expressed by *Escherichia coli* as eXact-tag fused protein (Bio-Rad), and purified by Profinity eXact Fusion-Tag system (Bio-Rad). The eluted protein was further purified by Superdex75 (GE healthcare) in TBS.

#### Preparation of h1F3Fab fragment

The Fab fragment of h1F3 was generated by papain digestion, and the Fc region was removed by passing through HiTrap Protein A HP (GE Healthcare). The flow-through fractions were dialyzed against acetate-Na, pH 5.0, and purified with Mono S (GE Healthcare) by linear pH gradient to phosphate-Na, pH 6.5. The main eluate was finally purified by Superdex200 (GE Healthcare) in TBS.

#### Preparation of the h1F3 Fab-PcrV (130–263) complex

The Fab fragment of h1F3 and PcrV (130–263) was mixed at a molar ratio of 1:2 in TBS containing 0.5-mM of Tris(2-carboxyethyl)phosphine (TCEP) at 4°C, overnight. The equimolar protein complex was separated by Superdex200.

#### Crystallization of h1F3 Fab-PcrV (130–263) complex

The protein solution of 8 mg/mL (10-mM Tris-Cl, 30-mM NaCl, and 1-mM TCEP) was used for the crystallization. Optimal crystals were obtained by the sitting drop vapor diffusion method, mixing an equal volume of the reservoir solution containing 0.1-M acetate-Na, pH 5.6, 24% (wt/vol) PEG3350, and 0.125-M CaCl_2_, grown at 20°C over a week. The crystals were cryoprotected by soaking in the reservoir solution with 18% (vol/vol) glycerol.

#### X-ray data collection and structure determination

Diffraction images were collected by MicroMax-007 HF X-ray generator. Data were processed using HKL2000. Initial phases were obtained by molecular replacement with MOLREP, using the m1F3 Fab sole structure (in house data) and the LcrV (PDB ID: 1R6F) structure, which is a PcrV homolog of *Yersinia pestis*, as search models. Subsequent model building and refinement were done with ARP/wARP, PHENIX refmac, and COOT. The overall structure was refined at 2-Å resolution. The data collection and refinement statistics are shown in Table S2.

### Cytotoxicity assay

U937 cell suspension, COT-143 solution, and bacterial suspension were mixed to make a final volume of 100 µL (8:1:1). The viable cell count was adjusted to 5 × 10^4^ cells per well. The optical density at 625 nm (OD_625_), of bacterial suspension was adjusted to 0.005. The multiplicity of infection was approximately 1. D-PBS, instead of test substance, and assay medium, instead of bacterial suspension, were used as controls if needed. The mixtures were incubated at 37°C for 4 hours in a humidified atmosphere of 5% CO_2_. After the incubation, 50 µL of the supernatants was mixed with an equal volume of CytoTox 96 substrate mix (Promega) dissolved in assay buffer and incubated at room temperature for 10–30 min for lactose dehydrogenase (LDH) measurement. After the incubation, Abs492 values were measured for LDH amount determination.

### Neutralization assay

U937 cell suspension, COT-143 solution, rPcrV solution, and bacterial suspension were mixed to make a final volume of 100 µL (7:1:1:1). The viable cell count was adjusted to 5 × 10^4^ cells per well. The OD_625_ of the bacterial suspension was adjusted to 0.005. COT-143 solution and rPcrV were mixed and pre-incubated at room temperature for more than 15 min. The rPcrV amount was 0.3-, 1.0-, 3.0-, and 10-fold that of the test substance in molar ratio. The mixtures were incubated at 37°C for 4 hours in a humidified atmosphere of 5% CO_2_. After the incubation, 50 µL of the supernatants was mixed with an equal volume of CytoTox 96 substrate mix dissolved in assay buffer and incubated at room temperature for 10–30 min for LDH measurement.

### Mouse pneumonia model

*P. aeruginosa* PA103 was cultured overnight at 37°C in MHB. The bacterial suspension was diluted with D-PBS to approximately 10^7^ CFU/mL and finally mixed with 5% gastric mucin to approximately 10^6^ CFU/mL (bacterial suspension: 5% gastric mucin = 1:9). Mice were anesthetized by intramuscular injection of the mixture of butorphanol, medetomidine, midazolam, and saline (10:3:8:79 as volume). The administration volume of the mixture was approximately 0.1 mL/mouse. The mice were infected by intranasal instillation of bacterial suspension. Inoculation volume was 0.07 mL/mouse. COT-143 was administered intravenously 24 hours before infection. Ceftazidime was subcutaneously administered 2 hours after infection.

### Viable bacterial counts in lungs

The lung was removed from surviving and dead mice and then homogenized in MHB. The homogenates were serially diluted 10-fold with MHB. These dilutions were pipetted into brain heart infusion agar (BHIA) containing 0.1% (wt/vol) KNO_3_. After approximately 42 hours of incubation at 37°C, the numbers of colonies grown in agar plates were counted. The number of CFU in the plate showing 20–300 was adopted for calculation.

### Determination of cytokine levels in lungs

The lung homogenates of the non-infected group and the remaining lung homogenates of the infected groups after the viable bacterial counts were centrifuged and filtrated to collect supernatants. The supernatants were stored at −80°C until measurement. Determinations of MIP-2, IL-6, and IL-1β concentrations in the supernatant were measured by using the Quantikine ELISA kit (R&D Systems) following the instructions. The samples were diluted with D-PBS before measurement. All the samples were prepared in duplicate. The average quantity of cytokine concentration was calculated from these two measurement values.

### Contact-dependent hemolysis assay

Sheep RBCs (Nippon Biotest Laboratories Inc.) were washed with RPMI1640 supplemented with 10% fetal bovine serum until the supernatant became clear and were then condensed to 1 × 10^9^ cells/ mL. Bacterial overnight culture (95 µL) and 20× antibody solution (10 µL) were mixed in a round-bottom 96-well plate. After addition of RBC (95 µL), centrifugation was carried out at 2,000 rpm for 10 min at room temperature, and the samples were incubated at 37°C. The sample solution was suspended and centrifuged at 2,000 rpm for 10 min at room temperature. The amount of hemoglobin in supernatant was determined by measuring Abs450. SDS of 0.1% was used for full hemolysis. Hemolysis activity is calculated as follows:


Hemolysis (%)={(X−B)/ (F−B)}×100

where *X* is the measured Abs450 value of samples; *B* is the Abs450 value of the background; and *F* is the Abs450 value of full hemolysis by SDS.

### Preparation of anti-PopB and anti-PopD mouse antibodies

Recombinant PopB and PopD proteins were expressed by *E. coli* BL21(DE3)/pET15 as N-term 6× poly His form. Each recombinant protein was collected in insoluble fraction. Each insoluble protein was denatured in 6-M guanidine-HCl and was purified by a Ni-NTA Superflow under denatured condition. The purified protein was refolded to phosphate-buffered saline (PBS) by dialysis. Anti-PopB antibody was isolated by panning with MorphoSys HuCal phage libraries against the immobilized PopB protein. The isolated anti-PopB Fab was cloned into mouse IgG heavy- and light-chain vectors and produced as recombinant IgG using FreeStyle 293 Expression System (Thermo Fisher Scientific). Anti-PopD antisera was generated by conventional mouse immunization by four times intraperitoneal injection of the 1:1 (vol/vol) mixture of 30-µg PopD and Freund’s adjuvant at intervals of 3 weeks.

### RBC membrane preparation after contact hemolysis

The procedure described below is mainly based on references 11 and 12. RBC, 100× protease inhibitor cocktail, and bacterial suspension were mixed. Centrifugation was carried out at 2,000 rpm for 10 min at 4°C followed by incubation for 1 hour at 37°C. All RBCs were lysed with ice-cold distilled water. After addition of ice-cold 62% sucrose-Tris-saline (final concentration is 46% sucrose), the sample was deposited at the bottom of a centrifuge tube and layered with 44% and then 25% sucrose-Tris-saline. Centrifugation was carried out at 15,000 × *g* for 16 hours at 4°C. Membrane fraction at the 25%/44% interface was taken, and membrane was collected by ultracentrifugation at 450,000 × *g* for 20 min. Membrane pellets were dissolved in 2% SDS-Tris-saline. The solution was mixed with SDS sample buffer containing dithiothreitol (DTT) and incubated for 5 min at 95°C.

### Western blotting analysis

The denatured proteins of RBC membrane were separated at 100 V for 90–120 min in Bis-Tris gradient gel and transferred onto polyvinylidene difluoride (PVDF) membranes. The membranes were blocked with 2% ECL prime blocking reagent for 2 hours and incubated overnight at 4°C with anti-PopB mouse monoclonal antibody or PopD mouse antisera diluted 1:1,000. Antibody binding was detected with horse radish peroxidase (HRP)-conjugated goat anti-mouse IgG diluted 1:5,000 followed by detection of HRP chemiluminescence emission using LAS3000 system (GE Healthcare).

### Preparation of neutrophils from mouse

Mice were treated with 10% casein (1 mL/mouse) by intraperitoneal injection twice. The second injection was performed 20 hours after the first injection. The mice were anesthetized by intramuscular injection of the mixture of butorphanol, medetomidine, midazolam, and saline (10:3:8:79 as volume, 0.1 mL/mouse) 4 hours after the second injection. Hank’s balanced salt solution without calcium/magnesium [HBSS (−)] containing 0.1% gelatin (5 mL/mouse) that was warmed at 37°C was injected into the peritoneal cavity of anesthetized mice. The injected solution was collected from the peritoneal cavity. The collected solution was centrifuged (3,000 rpm, 5 min, 20°C). The collected pellet containing neutrophils was washed once with HBSS (+) containing 0.1% gelatin by centrifugation (3,000 rpm, 5 min, 20°C). Neutrophil suspension was adjusted to approximately 7.0 × 10^7^ cells/mL.

### Phagocytosis assay

*P. aeruginosa* PA103 was grown in MHB for approximately 20 hours at 37°C with constant shaking at 150 rpm. The culture medium of the bacterial suspension was replaced with HBSS (+) containing 0.1% gelatin by centrifugation and adjusted to approximately 7.0 × 10^6^ CFU/mL. This adjusted suspension was used as the bacterial inoculum. Determination of viable bacterial counts of adjusted suspension was performed in duplicate. The bacterial suspension, mouse serum, and test substance solution [COT-143 in HBSS (+) containing 0.1% gelatin] were mixed in the presence or absence of cytochalasin D (final concentration of 0.5 µg/mL) and incubated at 37°C for 30 min using a rotary shaker at 150 rpm followed by the addition of the 60 µL of neutrophil suspension. The 600 µL of the test solution was incubated at 37°C for 3 hours using a rotary shaker at 150 rpm. All the samples were performed in triplicate.

Determination of viable bacterial counts was performed 3 hours after initiation of the assay as follows: 50 µL of the samples was mixed with 450 µL of D-PBS containing 10% saponin and then incubated for 5 min at 37°C to disrupt neutrophils. The samples were serially diluted 10-fold with MHB. Aliquots of 0.1 mL of these dilutions were pipetted into BHIA containing KNO_3_. After 24–72 hours of incubation at 37°C, the number of colonies grown in agar plates was counted. The number of CFUs in the plate showing 20–300 was adopted for calculation. When there were two plates showing 20–300 CFU in each sample with serial dilution, the mean of both numbers in the plates was adopted. When the lowest dilution plate showed 5–19 CFU or the highest dilution plate showed >300 CFU, the actual number of CFU in the plate was adopted for calculation. When the lowest dilution plate showed 0–4 CFU, 5 CFU was adopted for the calculation.

Determination of survival rate of neutrophils was performed 3 hours after initiation of the assay as follows: 50 µL of the samples was mixed with 125 µL of 0.1% gelatin-containing HBSS (−) containing 0.1% gelatin. The diluted samples of 50 µL were mixed with 50 µL of trypan blue solution. Ten microliters of the mixture was applied to the cell counter plate (Watson), followed by counting of the unstained surviving cells. Survival rate was calculated as follows:


Survival rate (%)=(X/Y)×100

where *X* is the number of surviving cells in the sample, and *Y* is the initial number of cells.

## Data Availability

The data that support the findings of this study are available in the supplemental material of this article. The DNA sequence data used in this study were based on the Pseudomonas Genome Database (https://www.pseudomonas.com/). The crystal structure of the complex between h1F3 Fab and PcrV fragment and its data files have been deposited with the Protein Data Bank (PDB) under accession number 9JBQ.

## SUPPLEMENTAL MATERIAL

Supplemental material aac.00694-24-s0001.docx Tables S1 and S2; Fig. S1.

ASM does not own the copyrights to Supplemental Material that may be linked to, or accessed through, an article. The authors have granted ASM a non-exclusive, world-wide license to publish the Supplemental Material files. Please contact the corresponding author directly for reuse.
