# Supplementary material for: Novel humanized anti-PcrV monoclonal antibody COT-143 protects mice from lethal Pseudomonas aeruginosa infection via inhibition of toxin translocation by the type III secretion system
Source: Antimicrob Agents Chemother. 2024 Sep 13;68(10):e00694-24. doi: 10.1128/aac.00694-24 (PMC11459929; doi:10.1128/aac.00694-24)
Supplement: Supplemental material — Tables S1 and S2; Fig. S1. [file aac.00694-24-s0001.docx]

**Supplementary Table 1. All interactions between the CDR3 regions of h1F3 and PcrV residues**

| h1F3 residue | | PcrV residue | Interaction |
| --- | --- | --- | --- |
| CDR heavy chain 3 | ^96^Gly | ^199^Asp | Hydrogen bond |
|  | ^97^Asn | ^209^Asp | Hydrogen bond |
|  | ^100a^Tyr | ^209^Asp | Hydrogen bond |
|  |  | ^212^Ser | Hydrogen bond |
|  | ^100b^Ty | ^226^Asp | Hydrogen bond |
| CDR light chain 3 | ^91^Trp | ^199^Asp | Hydrogen bond |
|  | ^92^Arg | ^199^Asp | Hydrogen bond |
|  |  | ^201^Phe | Hydrogen bond |
|  | ^94^Tyr | ^204^Lys | Hydrogen bond |
|  |  | ^170^Asp | Hydrogen bond |

**Supplementary Table 2. Crystallographic data collection and refinement statistics**

| Data collection |  |
| --- | --- |
| Wavelength (Å) | 1.5418 |
| Resolution (Å) | 50-2.0 |
| Space group | *P1* |
| Unit Cell |  |
| *a, b, c* (Å) | 44.7 52.0 70.0 |
| *α, β, γ* (º) | 99.0 104.6 115.2 |
| Number of observations | 125659 |
| Unique reflections | 33530 |
| Redundancy | 3.7(3.7) |
| Completeness (%) | 94.9(90.9) |
| R_merge_ (%) | 7.4 (32.2) |
| Refinement statistics |  |
| Resolution limits (Å) | 23.5-2.0 (2.1-2.0) |
| No. of reflections | 33563 (2939) |
| No. of test set reflections | 1697 |
| R_cryst_ /R_free_ (%) | 16.9/21.6 (19.0-26.6) |
| Average B factor (Å^2^) | 22.7 |
| Rms deviations |  |
| Bond lengths (Å) | 0.007 |
| Bond angles (°) | 1.136 |
| Number of atoms |  |
| Protein | 4261 |
| Water | 309 |
| Ramachandran plot |  |
| Favored (%) | 98 |
| Allowed (%) | 2 |
| Outlier (%) | 0 |


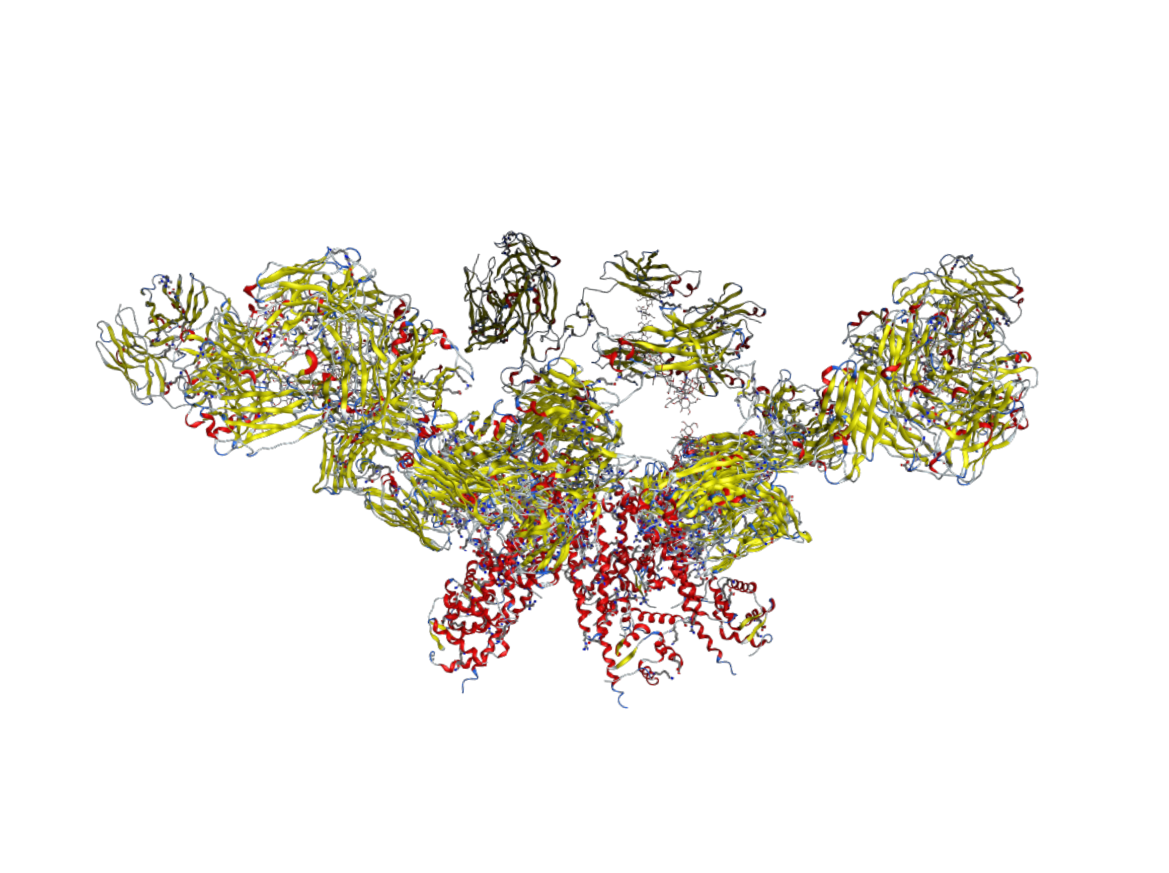


**Supplemental Figure 1.** Oligomeric structure of PcrV. Manually modeled oligomeric structure of the PcrV-h1F3 based on the scanning transmission electron microscopy structure of needle and its tip complex of LcrV in Yersinia enterocolitica that is an orthologue of PcrV, presented in Molecular Microbiology (2007) 65(5), 1311–1320.
